# Ultra-Fast Electrochemical Expansion for Rapid Enhancement of Graphite Paper Electrode

**DOI:** 10.1021/acsphyschemau.5c00014

**Published:** 2025-06-12

**Authors:** Praeploy Chomkhuntod, Sukanlaya Kornnum, Sirintra Arayawate, Bin Wang, Pawin Iamprasertkun

**Affiliations:** † School of Bio-Chemical Engineering and Technology, Sirindhorn International Institute of Technology, 37698 Thammasat University, Pathum Thani 12120, Thailand; ‡ Research Unit in Sustainable Electrochemical Intelligent, 37698 Thammasat University, Pathum Thani 12120, Thailand; § School of Integrated Science and Innovation, Sirindhorn International Institute of Technology, 37698 Thammasat University, Pathum Thani 12120, Thailand; ∥ State Key Laboratory of Heavy Oil Processing, College of Chemistry and Chemical Engineering, China University of Petroleum (East China), Qingdao 266580, P. R. China

**Keywords:** Graphite Expansion, Acid Treatment, Supercapacitor, Sustainable Energy Storage, Electrode

## Abstract

Graphite paper is widely used in energy storage systems such as batteries and supercapacitors due to its availability, cost, and excellent chemical and thermal stability. However, its hydrophobic surface and dense structure limit electrolytic ion diffusion in aqueous supercapacitors, reducing electrochemical performance. To address these issues, graphite paper was treated with mild acid solution and electrochemically oxidized for varying durations to enhance its properties for high-performance supercapacitors. Acid treatment not only expanded the graphite but also functionalized its surface, making it more hydrophilic, as confirmed by FTIR and contact angle measurements. This modification improved the electrode’s electrochemical performance by facilitating better ion diffusion and insertion, resulting in increased specific capacitance. After 5 min of treatment, the graphite layers enlarged from 35 to 469 μm, resulting in an enhanced specific capacitance of 219 F g^–1^ at 10 mV s^–1^ but poor cycling stability with 80% capacitance retention after 500 cycles. In contrast, the 3 min treatment achieved a specific capacitance of 113 F g^–1^ with excellent cycling stability without significant capacitance fading. These results highlight the importance of optimizing both the structural and chemical properties of graphite for achieving high performance and long cycling stability.

## Graphical abstract

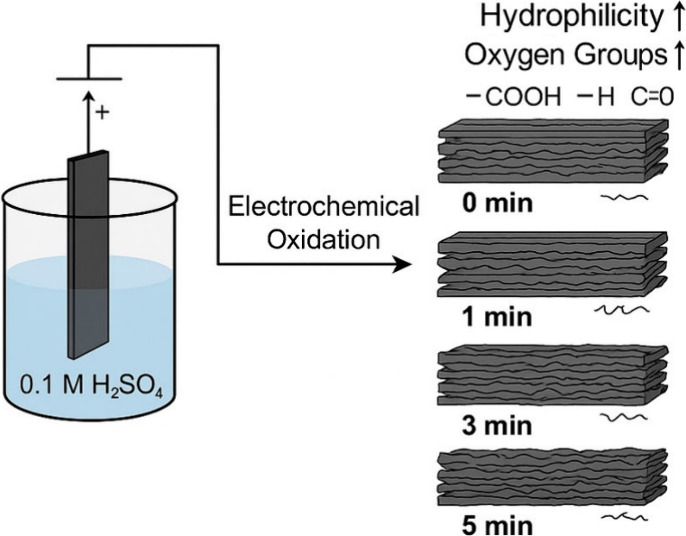

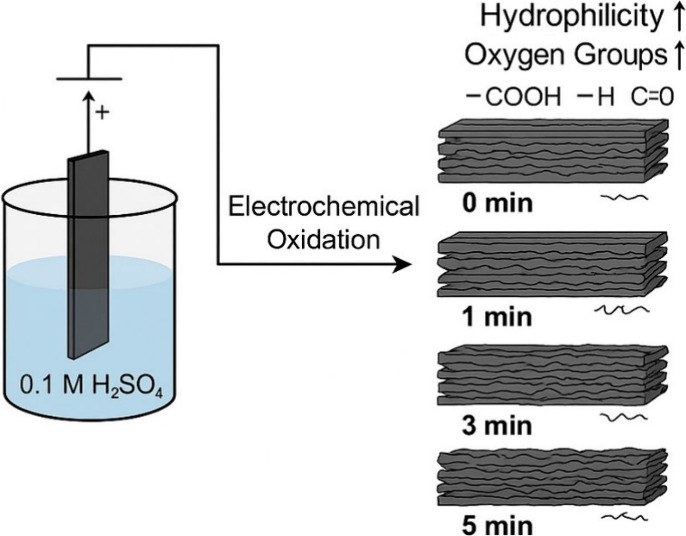

## Introduction

Carbon-based materials are extensively used in electrochemical energy storage due to their exceptional properties, including high electrical conductivity, large surface area, and both chemical and electrochemical stability.^1^ Particularly, graphite offers many advantages such as low cost, natural abundance, and excellent chemical properties and high electrochemical performance, which can be attributed to its layered structure that facilitates ion diffusion and intercalation during charge storage.^2^ Additionally, graphite paper (GF) is a promising substrate for supercapacitors due to its high conductivity and mechanical flexibility. These properties arise from the strong π–π stacking interactions between the graphene layers, which enhance both electrical conductivity and mechanical strength.^3,4^ However, graphite has low surface area and hydrophobic nature which impedes its practical supercapacitor applications.^2,5,6^ To address these limitations, exfoliation has emerged using various techniques such as chemical, thermal, and electrochemical processes.^2,7–9^ Among them, chemical and electrochemical processes have been widely used due to scalability and the high yield of exfoliated/expanded graphite.^8^ This process involves ions intercalation between the graphite layers, leading to an expansion of the material.^10^ This exfoliation increases the surface area and generates more accessible sites through the enlarged interlayer spacing, which facilitates ion insertion, resulting in enhanced ion diffusion and specific capacitance.^2,5^ Furthermore, this treatment functionalizes the graphite surface by introducing oxygen-containing groups such as hydroxyl, carboxyl, and carbonyl, which not only enhance the hydrophilicity of the material but also improve its compatibility with aqueous electrolytes.^11^ These oxygen functionalities further contribute to redox reactions with electrolyte ions, thereby enhancing the specific capacitance through these redox processes.^12,13^

Several intercalation-based exfoliation techniques have been developed, particularly using strong acid solutions, such as concentrated H_2_SO_4_ and CH_3_COOH.^14,15^ These strong acids facilitate the intercalation of ions into the graphite structure, leading to its expansion and the formation of a more porous network. This increased porosity improves ion accessibility, facilitates ion diffusion, and enhances the material’s specific capacitance. However, the use of strong acids raises environmental concerns and can cause excessive oxidation or structural damage, weakening the structural integrity of the graphite and reducing its long-term cycling stability. Therefore, the challenge is to explore a more sustainable, safer, and practical method for graphite expansion.

In recent years, mild acid solutions combined with electrochemical treatment have emerged as effective methods for expanding graphite, particularly for creating few-layer graphene structures. These methods are advantageous over traditional techniques, as they result in minimal oxidation and maintain the structural integrity of the graphite material.^2,16–18^ In this work, we explore the use of a mild acid solution (0.1 M H_2_SO_4_) in combination with electrochemical oxidation at +10 V to expand graphite paper, with the goal of optimizing the expansion process while minimizing excessive oxidation and exfoliation. By minimizing oxidation from concentrated acid solutions, our approach effectively preserves the graphite’s layered structure while enhancing its surface functionality, flexibility, and overall performance. Surface functionalization and wettability were characterized using FTIR and contact angle measurements, while morphological changes due to the graphite expansion were investigated through SEM and BET surface area analysis. This approach not only improves the electrochemical properties of graphite but also offers a safer, more sustainable alternative to conventional graphite expansion methods.

## Results and Discussion

Overall, the graphite paper electrodes were successfully expanded through electrochemical oxidation in a mild 0.1 M H_2_SO_4_ solution at varying oxidation times. This approach enhances the structural, electrical, and electrochemical properties of the substrate^19^ (i.e., graphite papers), making them a viable alternative current collector capable of storing charge during electrochemical processes. The morphological changes in the expanded graphite were analyzed using SEM (Figure 1). Initially, the untreated graphite exhibited a smooth, uniform surface with well-aligned layers and a compact layered structure (Figure S1), with a thickness of approximately 35 μm (Figure 1a). After treated for 1 min, slight surface etching and increased roughness can be observed, with the thickness increasing to 113 μm (Figure 1b). As the treatment time increases, the graphite surface becomes rougher, with the formation of micropores, while the interlayer spacing further expands, resulting in a thickness of 245 μm after 3 min treatment (Figure 1c). Furthermore, after 5 min, surface etching and layer expansion are more pronounced, with a significant increase in thickness to 469 μm (Figure 1d). This increase in thickness, along with the expansion of interlayer spacing and graphite expansion, can be attributed to ion intercalation during electrochemical acid treatment, owing SO_4_^–2^ ion insertion and the formation of oxygenated functional groups on the graphite surface.^20^ These functional groups disrupt the π-conjugated electron system of graphite, leading to an increase of the energy gap between oxygen atoms bonded to carbon and reducing conductivity.^21^ However, this expanded graphite structure offers additional space for ion insertion and ion diffusion, leading to improved capacitance and rate performance of the graphite electrodes. This is well aligned with the X-ray diffraction (XRD) in Figure 1e. The XRD patterns of all samples show the characteristic diffraction peak at approximately 26°, which corresponds to the (002) plane (JCPDS 76-1621), with an interlayer distance of about 0.33 nm in the pristine graphite.^15,22^ After electrochemical treatment, the interlayer distance increases slightly, as evidenced by the shift in the diffraction peak. Specifically, as the electrochemical expansion time increases, the interlayer spacing changes from 3.33 Å in GP to 3.37, 3.38, and 3.39 Å for EGP-1, EGP-2, and EGP-3, respectively.^23^ These results indicate the successful intercalation and expansion of the graphite structure. Despite these changes, the basal graphite structure remains largely intact in both the pristine and electrochemically expanded graphite materials, confirming that the graphite preserves its layered structure after electrochemical treatment. In addition to the peak shift, the broadening of the (002) peak is observed. The broadening becomes more pronounced with longer electrochemical expansion times and can be attributed to the intercalation process.^24^ During the expansion process, sulfate ions (SO_4_^2–^) are inserted between the graphite layers, causing the interlayer distance to expand and leading to peak broadening due to increased stacking disorder. The broad humps observed at the (002) position suggest the presence of a residual graphite sulfate phase, providing further evidence of SO_4_^2–^ ion intercalation into the graphite layers.^22^ This effect becomes more pronounced with longer electrochemical treatment times due to sufficient duration for the sulfate ions to be effectively inserted. Note that the full XRD pattern can be found in Figure S2. Moreover, the specific surface area of all samples was measured using N_2_ adsorption through BET analysis. As the graphite sheets expand and their thickness increases, the surface area of the expanded graphite papers significantly increases, rising from 28 m^2^ g^–1^ for the pristine graphite paper (GP) to 126 m^2^ g^–1^ for EGP-1, 217 m^2^ g^–1^ for EGP-2, and 586 m^2^ g^–1^ for EGP-3, as shown in Figure S3. The increasing surface area directly improves the electrochemical active area of the graphite electrodes by surface charges stored. Therefore, the larger surface area of the graphite electrode allows for more ions to be stored, which significantly enhances the capacitance of the electrode.^25,26^ Furthermore, the electrical conductivity was measured using 4-point probe measurements, and the results were plotted against the electrode thickness, as shown in Figure 1f. As expected, pristine graphite paper (GP) exhibited the highest conductivity, owing to its well-ordered graphite structure and π-conjugated network, which facilitates electron transfer along the basal planes.^27^ However, with increasing electrochemical treatment time, the electrical conductivity progressively decreased due to the extent of surface functionalization and oxidation, resulting in the energy gap in the electron density of states between oxygen atoms bonded to carbon, making it lower conductivity.^28^ Furthermore, the formation of functional groups on the graphite surface significantly impedes conductivity by introducing localized charge trapping sites.^29,30^ Although this reduction in electrical conductivity might limit the electrode’s conductivity and electron transfer kinetics, the enhanced surface functionalization can improve the wettability and electrochemical reactivity of the electrodes by providing more active sites for redox reactions, which can enhance the overall performance of the graphite electrodes.^31^ Additionally, the thickness has a significant impact on their electrical conductivity, as thicker graphite electrodes exhibit lower conductivity due to increased internal resistance from the longer electron transport paths.^29^

**Figure 1:**
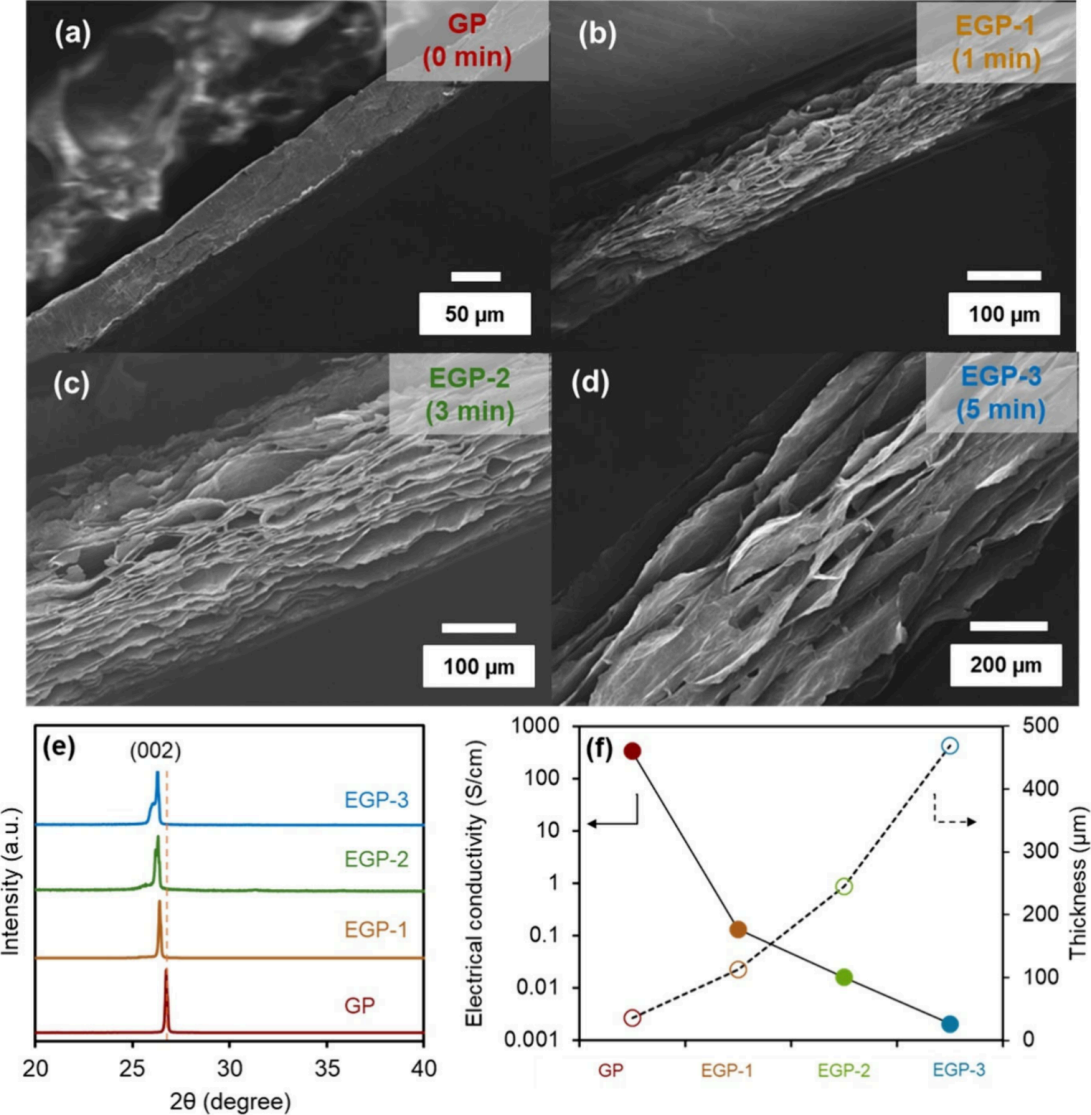
Structural and electrical properties after electrochemical expansion, showing the SEM images of (a) graphite paper, (b) 1 min, (c) 3 min, and (d) 5 min expansion, (e) the XRD patterns, and (f) electrical conductivity and thickness of the as-expanded electrodes.

These observations were confirmed by FTIR analysis and wettability measurements, as shown in Figure 2. Obviously, the new peaks appear in FTIR spectra at the range of 1744 to 1002 cm^–1^, indicating surface oxidation of the graphite due to the formation of various oxygenated functional groups, such as carboxylic acid (−COOH) groups^32^ after electrochemically expansion (see Figure 2a). This is confirmed by the band at 1744 cm^–1^, which corresponds to the carbonyl (C=O) stretching vibration, along with the peaks at 1477 cm^–1^ and 1278 cm^–1^, which are attributed to the stretching vibrations of −OH and −C–O groups, respectively.^33–35^ The intensity of these peaks increases with prolonger electrochemical treatment, suggesting that the formation of functional groups and the oxidation of the graphite surface intensify as the electrochemical treatment time increases. The result suggests the influence of extended treatment times in promoting a more functionalized graphite surface. Yet, the surface functionalization of graphite electrodes plays a crucial role in significantly enhancing their wettability, as demonstrated by contact angle measurements using water droplets. Initially, untreated graphite paper (GP) exhibits a contact angle of 123° (Figure 2b), which indicates the hydrophobic surface, owing to the dominance C–C and π bond (hybridized sp^2^ bonding) in graphite, resulting in its low surface energy.^36,37^ After electrochemical treatment, the contact angle slightly decreases to 63°, 62°, and 60° for EGP-1, EGP-2, and EGP-3, respectively, after 1, 3, and 5 min of treatment (Figure 2c–e). The reduction in contact angle confirms a significant improvement in the hydrophilicity of the graphite surface. Notably, a 5 min electrochemical treatment shows sufficiency to achieve optimal wettability. This enhanced wettability correlates with the introduction of oxygen-containing functional groups, as confirmed by the FTIR results.^2,38^ These functional groups, such as −OH, C=O, and −COOH increase the polarity of the graphite surface, which facilitates stronger interactions between its surface and water molecules, enhancing the electrode–electrolyte interface interaction.^29,39^

**Figure 2:**
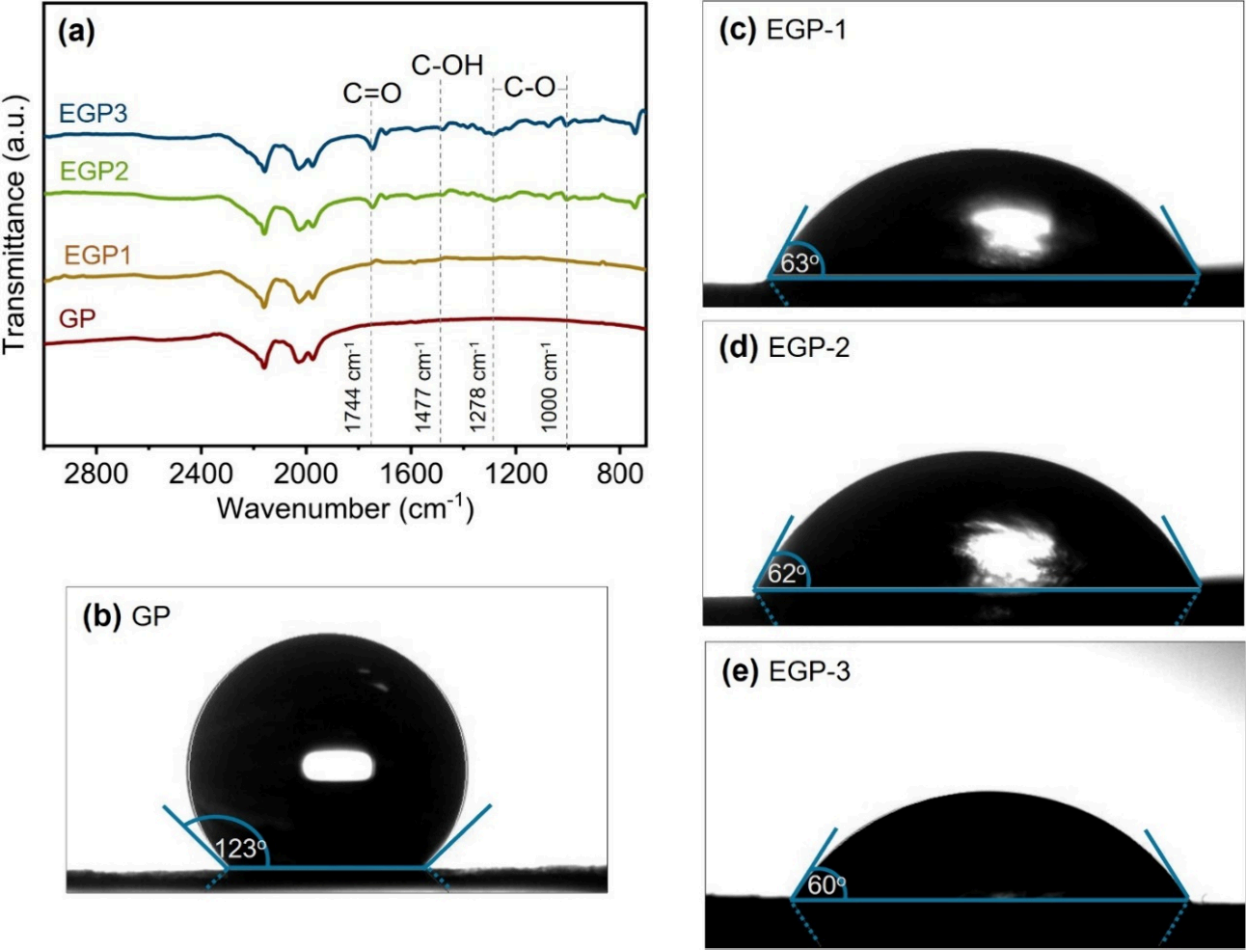
Surface functionality and wettability of the treated graphite papers. (a) FTIR spectra and the contact angle measurements of a water droplet on graphite paper treated at different treatment times: (b) 0 min-GP, (c) 1 min-EGP-1, (d) 3 min-EGP-2, and (e) 5 min-EGP-3.

To explore the effects of graphite expansion through electrochemical acid treatment on its electrochemical properties, cyclic voltammetry (CV) was primarily used along with electrochemical impedance spectroscopy (EIS). The CV measurements were measured between −0.4 and 0.8 V vs. Ag/AgCl at scan rates ranging from 10 to 100 mV s^–1^. Figure 3a compares the CV profiles of pristine graphite paper (GP) and expanded graphite papers at a scan rate of 50 mV s^–1^. The results show that pristine graphite exhibits a very small current response, suggesting negligible capacitive behavior, which can be attributed to its hydrophobic surface, which is incompatible for aqueous electrolyte.^40^ In contrast, the expanded graphite papers show a significantly larger CV response, suggesting improved charge storage capabilities.^41^ The area under the CV curve increases as the treatment time is extended, which reflects the enhanced electrochemical performance of the expanded graphite. For EGP-1 (1 min of expansion), the CV curve shows a regular rectangular shape, indicating that charge storage occurs mainly through electrostatic interaction on the surface.^42^ However, for EGP-2 (3 min) and EGP-3 (5 min), redox peaks are observed, indicating that redox reactions occur during charge and discharge between OH^–^ ions and acidic functional groups on the expanded graphite surface.^12,13,43^ EGP-3, in particular, shows more pronounced redox behavior with noticeable peak shifts, suggesting that ions are inserted into the graphite structure.^13,44^ In comparison, EGP-2 primarily stores charge through surface redox reactions facilitated by the functional groups. Moreover, the redox peaks of EGP-3 are observed at higher overpotentials compared to EGP-2, which can be attributed to differences in their structural and electronic properties.^45^ Although EGP-3 exhibits a larger *d*-spacing and higher surface area, which are generally favorable conditions for ion diffusion and charge storage, the shift to higher overpotentials is likely due to the higher oxygen-containing functional groups, resulting in reduced electrical conductivity at the redox-active sites, as supported by FT-IR and 4-point probe measurements.^46^ Additionally, the increased interlayer spacing may weaken interlayer electronic coupling, leading to an enhanced energy barrier for redox reactions.^47,48^ As a result, these phenomena contribute to the observed shift in redox peak positions, despite the improved morphological features of EGP-3.

**Figure 3:**
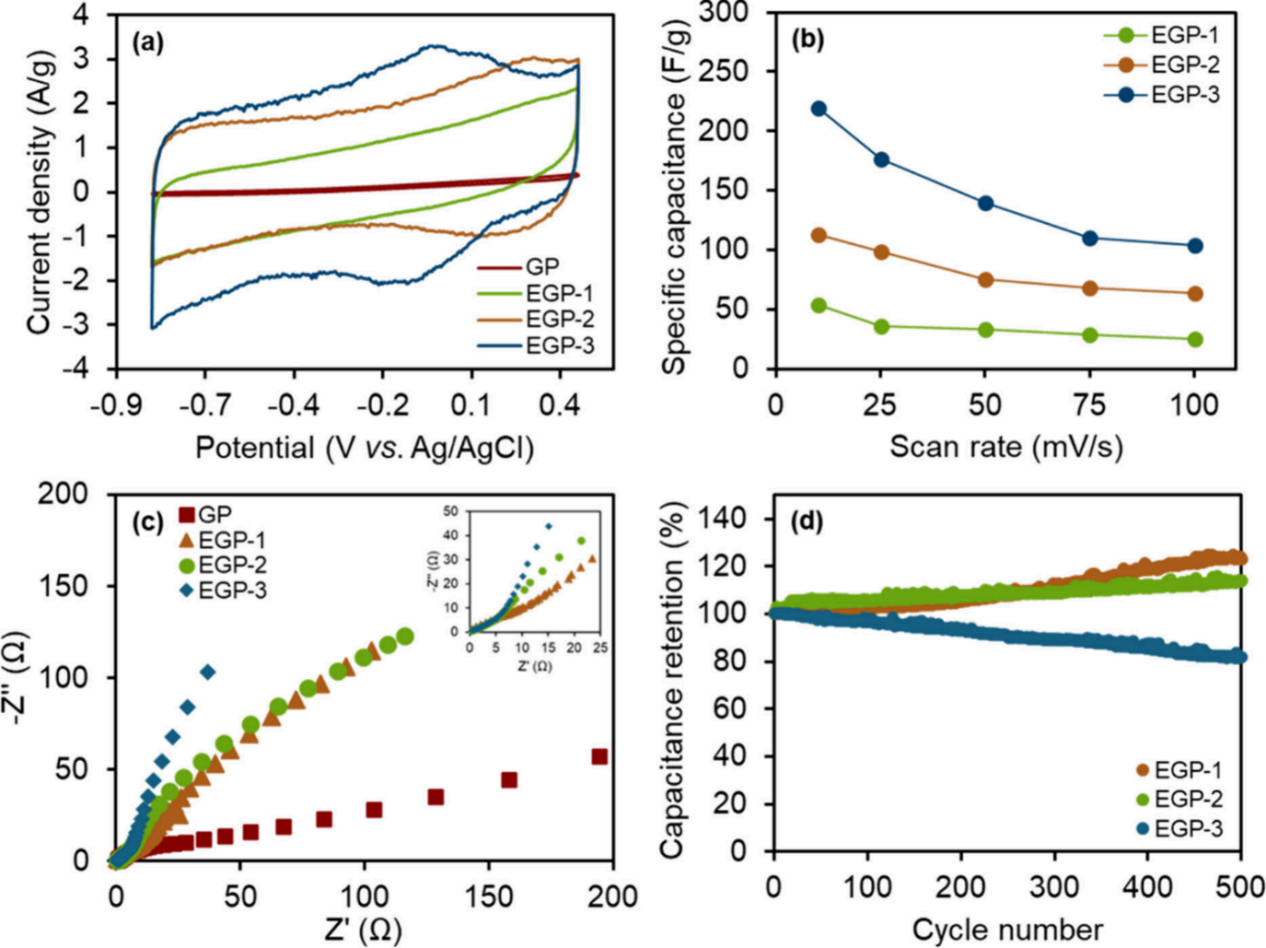
Electrochemical properties of the treated graphite papers. (a) CVs at 50 mV s^–1^. (b) Specific capacitance with respect to scan rates. (c) Nyquist plots. (d) Cyclic stability test.

In addition, the capacitive contribution and its capacitance of the prepared graphite electrodes was calculated from the CV data at various scan rates, as shown in Figure 3b. Since pristine graphite paper exhibits negligible capacitive response, it served only as a control for comparison. The specific capacitance of the expanded graphite papers was compared across different treatment times. The results clearly indicate that longer treatment times lead to higher specific capacitance. EGP-3 shows the highest capacitance at all scan rates, with values of 219, 177, 139, 110, and 104 F g^–1^ at 10, 25, 50, 75, and 100 mV s^–1^, respectively. EGP-2 shows a capacitance of 113 F g^–1^ at 10 mV s^–1^, while EGP-1 showed 54 F g^–1^ at the same scan rate. This improvement in capacitance can be attributed to the increased surface area and enhanced redox activity.^49^ EGP-3, which has the largest surface area and a more loosely structured layer arrangement, allows for better ion intercalation and diffusion, leading to the highest capacitance and better high-rate performance.^50^ In contrast, EGP-2, while still having a larger surface area and greater redox activity than EGP-1, shows higher specific capacitance because EGP-1 only stores charge through electrostatic ion adsorption.^42^ It should be noted that the noise observed in the CVs may be attributed to the low response current (<1 mA) as well as the relatively low electrical conductivity of the as-modified graphite papers. However, these issues could potentially be mitigated in future work through the use of a Faraday cage and current signal amplification.^51,52^

Moreover, electrochemical properties of the expanded graphite were further evaluated using EIS in the frequency range of 0.01 Hz to 100 kHz at the open circuit potential as illustrated in Nyquist plot in Figure[Fig fig3]c. *R*_s_ can be determined from the intercept of the high-frequency semicircle with the real axis, which corresponds to the ionic resistance of the electrolyte solution, the intrinsic resistance of electrode materials, and the contact resistance at electrode/electrolyte interface.^53,54^ All samples exhibit similar *R*_s_ values approximately 0.6–0.7 Ω, attributed to the comparable electrode materials and similar electrolyte during the measurements. All the expanded graphite electrodes display a near vertical line in the low-frequency region, indicating relatively low ion-diffusion resistance compared to the pristine graphite electrode (GP). This ion-diffusion resistance can also be called Warburg impedance (W), correlating to the ion-diffusion resistance in the bulk graphite electrode.^55^ Thus, a lower diffusion resistance of the expanded graphite (EGP) can be attributed to the improved wettability, larger surface area, and porous structure of the expanded graphite, which enhance the ion diffusion pathway. Notably, EGP-3 shows the most vertical line, indicating the fast ion diffusion.^12^ In the high-frequency region, the small semicircles are observed, corresponding to the charge transfer resistance (*R*_*ct*_) between the electrolyte and electrode.^15^ The *R*_*ct*_ for all the expanded graphite electrodes are likely the same (5–7 Ω), whereas the *R*_*ct*_ of the pristine graphite electrode (GP) is significantly higher (12 Ω). This difference can be attributed to the larger interlayer distance and the surface functional group of the expanded graphite electrodes, while the GP is incompatibility with the aqueous electrolyte leads to increased resistance between the electrolyte and the electrode.^12,56^ Overall, these findings suggest that ionic diffusion plays a more significant role in charge storage for the expanded graphite electrodes compared to charge transfer resistance, which typically found in graphite/graphene supercapacitors.^57^

Furthermore, the capacitive behavior from EIS measurements is further illustrated in Figure S5. As shown in the Bode phase plot (Figure S5a), EGP-3 exhibits the most ideal capacitive behavior, followed by EGP-2 and EGP-1, with phase angles of −72°, −62°, and −57°, respectively. Since a phase angle closer to −90° indicates more ideal capacitive characteristics, these values suggest a trend from more capacitive behavior in EGP-3 to less in EGP-1.^58,59^ Moreover, EGP-2 and EGP-3 demonstrate more pronounced redox behavior, while EGP-1 primarily exhibits electric double-layer capacitance (EDLC). This is consistent with the charge storage mechanisms observed in the CV measurements: EGP-1 stores charge mainly through electrostatic interactions on its surface, whereas EGP-2 and EGP-3 store charge via redox reactions. In addition, the capacitance derived from impedance data is plotted against frequency in Figure S5b, reflecting the actual accessible capacitance of the cell at different frequencies. Overall, the specific capacitance values obtained from EIS are in good agreement with those from CV and GCD measurements.^60,61^

Furthermore, the cycling stability of the as-prepared electrodes was evaluated at a scan rate of 50 mV s^–1^, as shown in Figure 3d. The improved capacitance observed for EGP-1 and EGP-2 is attributed to enhanced wettability over time. As an increase of cycle number, the electrolyte increasingly penetrates the graphite structure, facilitating improved ionic diffusion and transport within the electrode bulk.^58^ The results demonstrate that EGP-1 and EGP-2 exhibit excellent stability, with negligible capacitance loss over 500 cycles. In contrast, EGP-3 shows significant capacitance degradation, likely due to its overexpanded and unstable structure, as well as its charge storage mechanism involving ion intercalation and redox reactions, which can lead to structural changes and/or ion trapping during cycling.^62^

To gain deeper insights into the charge storage process of expanded graphite electrodes, the electrodes were cycled at various scan rates ranging from 10 to 100 mV s^–1^ to examine the contributions of capacitive-controlled (surface-controlled) and diffusion-controlled mechanisms. The capacitive contribution was determined by analyzing the relationship between the peak current (*i*) and scan rate (ν), as following the equations below:^63,64^

1 $$i=a\nu^{b}$$

2 $$i\left(\nu \right)=k_{1}\nu +k_{2}\nu^{0.5}$$

In these equations, *i* represents the current at peak potentials, and ν is the scan rate. The term *k*_1_ν represents the surface-controlled (capacitive) contribution, while *k*_2_ν^0.5^ corresponds to the diffusion-controlled mechanism. The *b*-value quantifies the relative contributions of the capacitive and diffusion-controlled processes. It can be determined by linearly fitting the slope of the log­(*i*) versus log­(v) plot, as shown in Figure 4a. Typically, electrode materials that exhibit both battery-type and capacitive charge storage have *b*-values between 1 and 0.5. A *b*-value of 0.5 signifies a diffusion-controlled process, whereas a *b*-value of 1 indicates a capacitor-controlled process. The *b-*value of EGP-1 is approximately 0.99, suggesting predominant capacitive charge storage at the electrode surface, whereas EGP-2 and EGP-3 exhibit *b*-values of 0.79 and 0.73, respectively, indicating greater contributions from the diffusion-controlled process due to ion intercalation, particularly EGP-3.^53^

**Figure 4:**
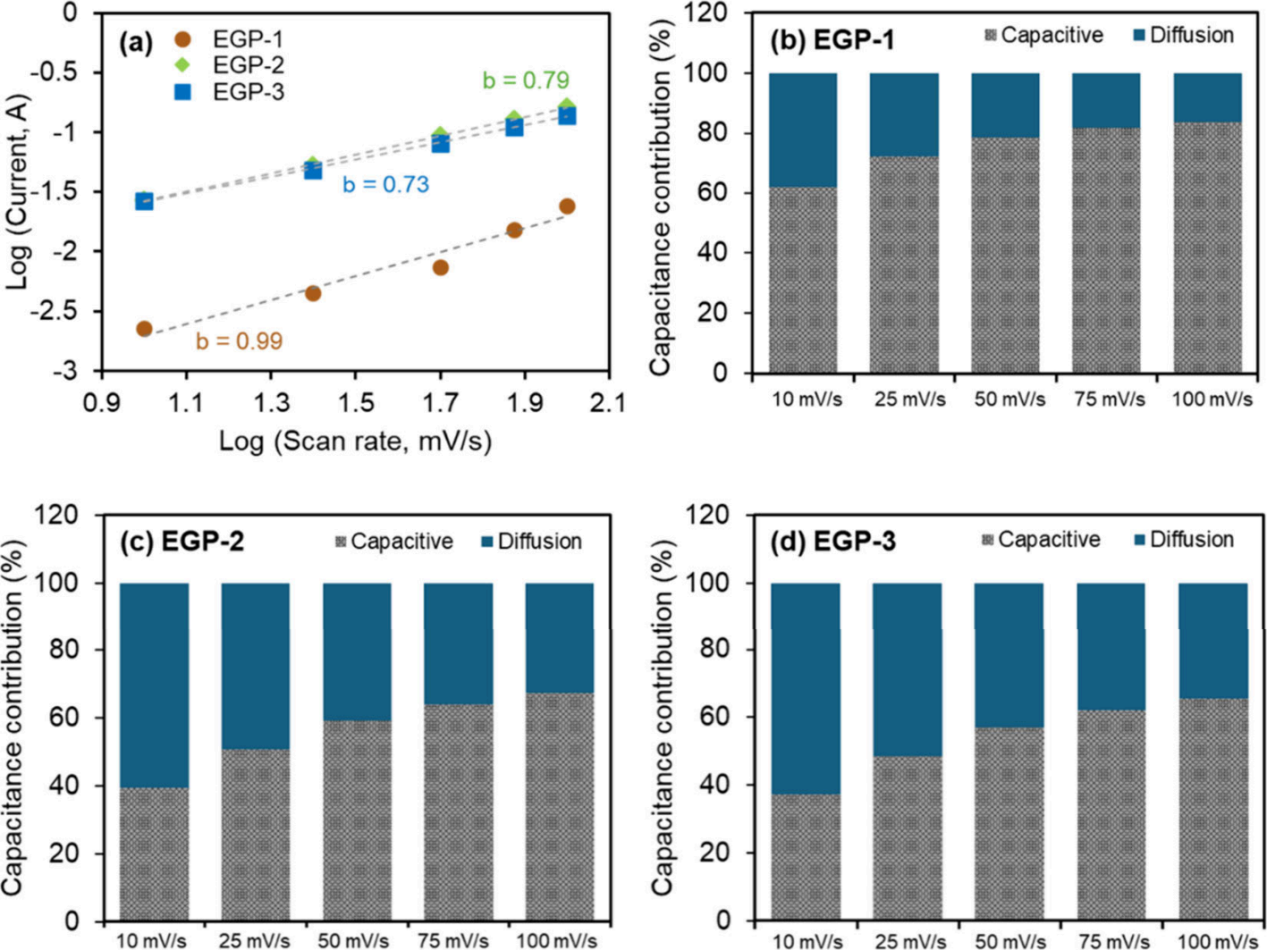
Analysis of capacitance contribution. (a) The plots of *b*-values used to determine the capacitive contribution. The calculated ratios of capacitive to diffusion-controlled contributions for (b) EGP-1, (c) EGP-2, and (d) EGP-3.

Figure 4b–d presents the distribution of capacitive and diffusion-controlled contributions for the expanded graphite electrodes prepared at different treatment times. The results show that the diffusion-controlled contribution becomes more prominent as the treatment time increases: EGP-1 < EGP-2 < EGP-3. For EGP-1, the surface-controlled contribution is dominant, as charge storage occurs through physical adsorption on the graphite surface via non-Faradaic processes.^65^ This is due to the presence of small amount of functional groups on its surface, as evidenced by the FTIR results. For EGP-2 and EGP-3, redox reactions are observed, especially for EGP-3 that stores charge through redox ion intercalation, which is diffusion-limited process.^66^ Generally, at higher scan rates, the capacitive contribution becomes more significant due to the limitations of ion diffusion in the electrolyte.^67^ Thus, the results confirm that the highest capacitance for EGP-3 is primarily attributed to ion intercalation during charge/discharge processes.^13^ Notably, even at a high scan rate of 100 mV s^–1^, more than 40% of the capacitance is retained from the diffusion-controlled mechanism, indicating the high-rate performance of the EGP-3, owing to its more accessible surface area and larger interlayer space within its loosely structured layers.^50^ These factors contribute to the enhanced specific capacitance and superior high-rate performance observed in EGP-3.

To further elucidate the impact of the electrochemical process in graphite exfoliation, a control experiment was conducted in which graphite paper was treated in an acid solution for 5 min without electrochemical assistance, as shown in Figure S6. The SEM images from both top-view and cross-sectional perspectives reveal negligible exfoliation and no noticeable change in thickness (Figure S6a,b). These results confirm that the electrochemical process plays a critical role in promoting efficient graphite exfoliation under mild acidic conditions. Additionally, the CV profiles of the graphite treated without electrochemical assistance exhibit no noticeable capacitive behavior (Figure S6c). In comparison, the graphite treated with electrochemical assistance displays a much stronger capacitive response. The difference between the two treatments can be attributed to the level of exfoliation achieved. Without the electrochemical process, the graphite is not effectively exfoliated, resulting in a lower surface area and limited electrochemical activity. Overall, the results suggest that our simplified electrochemical expansion in a dilute acid solution provides a safer, less toxic, and faster method to effectively exfoliate and oxidize graphite paper, offering a promising substrate for aqueous supercapacitors, as demonstrated in the benchmarking table in the Supporting Information (Table S1).

## Conclusions

This work demonstrates a safer and more facile method for expanding graphite using a mild 0.1 M H_2_SO_4_ acid solution through an electrochemical oxidation process at +10 V. This approach not only facilitates graphite expansion but also promotes surface functionalization, enhancing the wettability and redox activity of the graphite electrode. Due to the inherently hydrophobic nature of graphite, the introduction of oxygenated functional groups on its surface results in hydrophilic properties, which can improve the penetration and diffusion of electrolytic ions through the graphite structure. Additionally, the treatment time was optimized to maximize electrode performance. With increasing treatment time, the graphite structure was progressively expanded, and its surface was oxidized and functionalized. While expanded structures offer higher specific capacitance and improved rate performance due to increased surface area and accessibility, EGP-3 (treated for 5 min) exhibits instability during cycling, as evidenced by capacitance fading due to ion intercalation. In contrast, EGP-2 (treated for 3 min) shows excellent cycling stability over 500 cycles with high specific capacitance (113 F g^–1^ at 10 mV s^–1^), attributed to its surface redox reactions. Therefore, optimizing the treatment time and graphite structure is crucial for developing effective graphite electrodes for aqueous supercapacitors.

## Supplementary Material



## Notes

Experimental details including substrate preparation and electrochemical measurement and extended results containing SEM images, full pattern of XRD, N_2_ adsorption, and CVs at various scan rates (PDF)
