# Supplementary material for: Ultra-Fast Electrochemical Expansion for Rapid Enhancement of Graphite Paper Electrode
Source: ACS Phys Chem Au. 2025 Jun 12;5(4):318–26. doi: 10.1021/acsphyschemau.5c00014 (PMC12291138; doi:10.1021/acsphyschemau.5c00014)
Supplement: Supplementary file 1 [file pg5c00014_si_001.pdf]

Supporting Information

Ultra-Fast Electrochemical Expansion for Rapid Enhancement of Graphite Paper Electrode

Praeploy Chomkhuntod,[a,b] Sukanlaya Kornnum,[b, c] Sirintra Arayawate, [a,b] Bin Wang,[d]

Pawin Iamprasertkun,[a,b,]*

[a] School of Bio-Chemical Engineering and Technology, Sirindhorn International Institute of Technology, Thammasat University, Pathum Thani, 12120, Thailand (*pawin@siit.tu.ac.th*)

[b] Research Unit in Sustainable Electrochemical Intelligent, Thammasat University, Pathum Thani, 12120, Thailand

[c] School of Integrated Science and Innovation, Sirindhorn International Institute of Technology, Thammasat University, Pathum Thani, 12120, Thailand

[d] State Key Laboratory of Heavy Oil Processing, College of Chemistry and Chemical Engineering, China University of Petroleum (East China), Qingdao, 266580 P. R. China

Corresponding Author

*P. Iamprasertkun (email: pawin@siit.tu.ac.th) Tel: +66-2-986-9009 ext. 2306

https://orcid.org/0000-0001-8950-3330,

Keywords: Graphite Expansion; Acid Treatment; Supercapacitor; Sustainable Energy Storage

Experimental Section

Preparation of expanded graphite electrode

Graphite paper was expanded *via* electrochemical intercalation exfoliation. Initially, the 1x1 cm^2 graphite paper was immersed in 50 mL of 0.1 M H_2SO_4 solution for 1 h to ensure complete wetting. A potential of +10 V was then applied to the graphite paper using Chronoamperometry, with different dwelling times of 0, 1, 3, and 5 min. The samples were designated as GP (commercial graphite paper, 0 min), EGP-1 (1 min), EGP-2 (3 min), and EGP-3 (5 min). After electrochemical expansion, the graphite papers were thoroughly rinsed with DI water until the pH reached approximately 6-7, then dried at 80˚C for 24 h.

Material characterizations

The structural ordering was determined using a PANalytical X'Pert X-ray diffractometer with Cu Kα radiation at a wavelength of 1.5406 Å over a 2theta range of 10-60°. The morphology and surface features of the treated graphite were investigated using scanning electron microscopy (SEM, FEI/Philips XL30 E-SEM, Quanta 650), providing high-resolution images to observe the structural changes resulting from the treatments. The total specific surface area of the samples was determined by Brunauer-Emmett-Teller (BET) analysis, based on nitrogen adsorption-desorption measurements conducted at 77 K. Prior to the BET measurements, all samples were degassed at 150°C for 3 h to remove any adsorbed moisture or impurities. Electrical conductivity of the samples was measured using a four-point probe technique (JANDLE, Model RM3000+). The contact angle was measured using a Theta Optical Tensiometer (Biolin Scientific, Sweden) with OneAttension software. A water droplet

was placed on the sample surface, and the contact angle was calculated using Young's equation.[1]

Electrochemical measurement

The electrochemical performance of the as-expanded graphite papers was investigated using a three-electrode configuration with a Metrohm Autolab (PGSTAT302N). The as-prepared graphite electrodes were cut into 2x1 cm^2 pieces and used as the working electrodes, while an Ag/AgCl (3 M KCl solution) reference electrode and a platinum wire counter electrode were employed. The electrodes were tested in a 6 M KOH electrolyte solution. To control the active area, only a 1x1 cm^2 section of each graphite electrode was immersed in the electrolyte, and the electrodes were soaked in the solution for 30 minutes prior to electrochemical testing. Cyclic voltammetry (CV) and electrochemical impedance spectroscopy (EIS) were performed to investigate the electrochemical behaviour of the graphite electrodes. CV measurements were conducted within the voltage range of -0.8 to 0.4 V vs. Ag/AgCl at scan rates of 10 to 100 mV/s. EIS was carried out at open circuit voltage (OCV) with an amplitude of 10 mV, over a frequency range of 0.01 Hz to 100 kHz.

Results Section

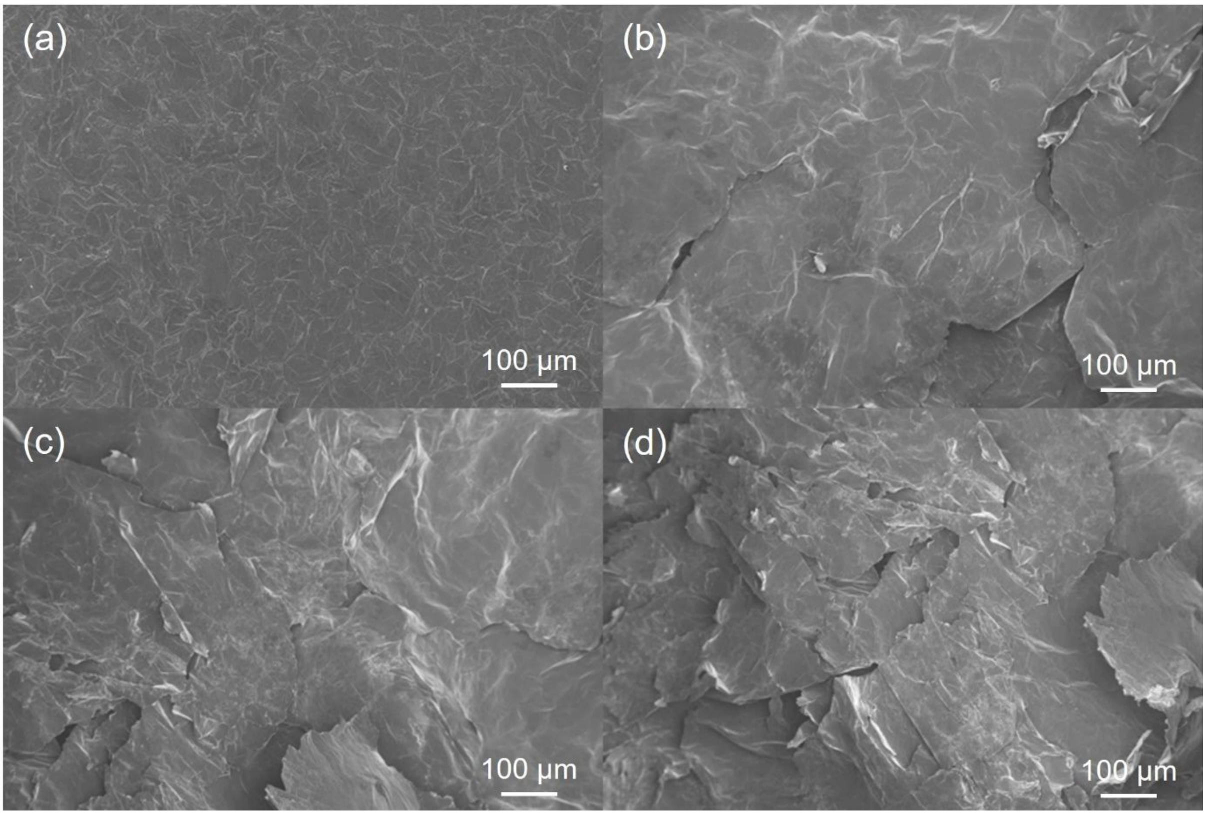

Figure S1. The surface morphology of the as-intercalated graphite papers (a) without modification, (b) 1 min, (c) 3 min, and (d) 5 min after electrochemical intercalation.

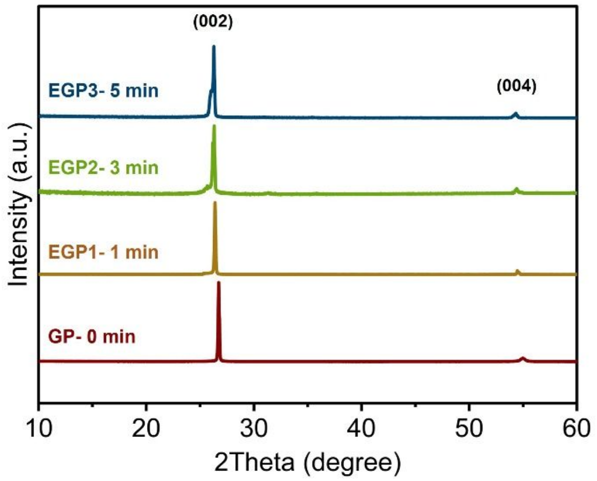

Figure S2. The XRD pattern of the as-electrochemically intercalated graphite papers, an extension to pattern in Figure 1.

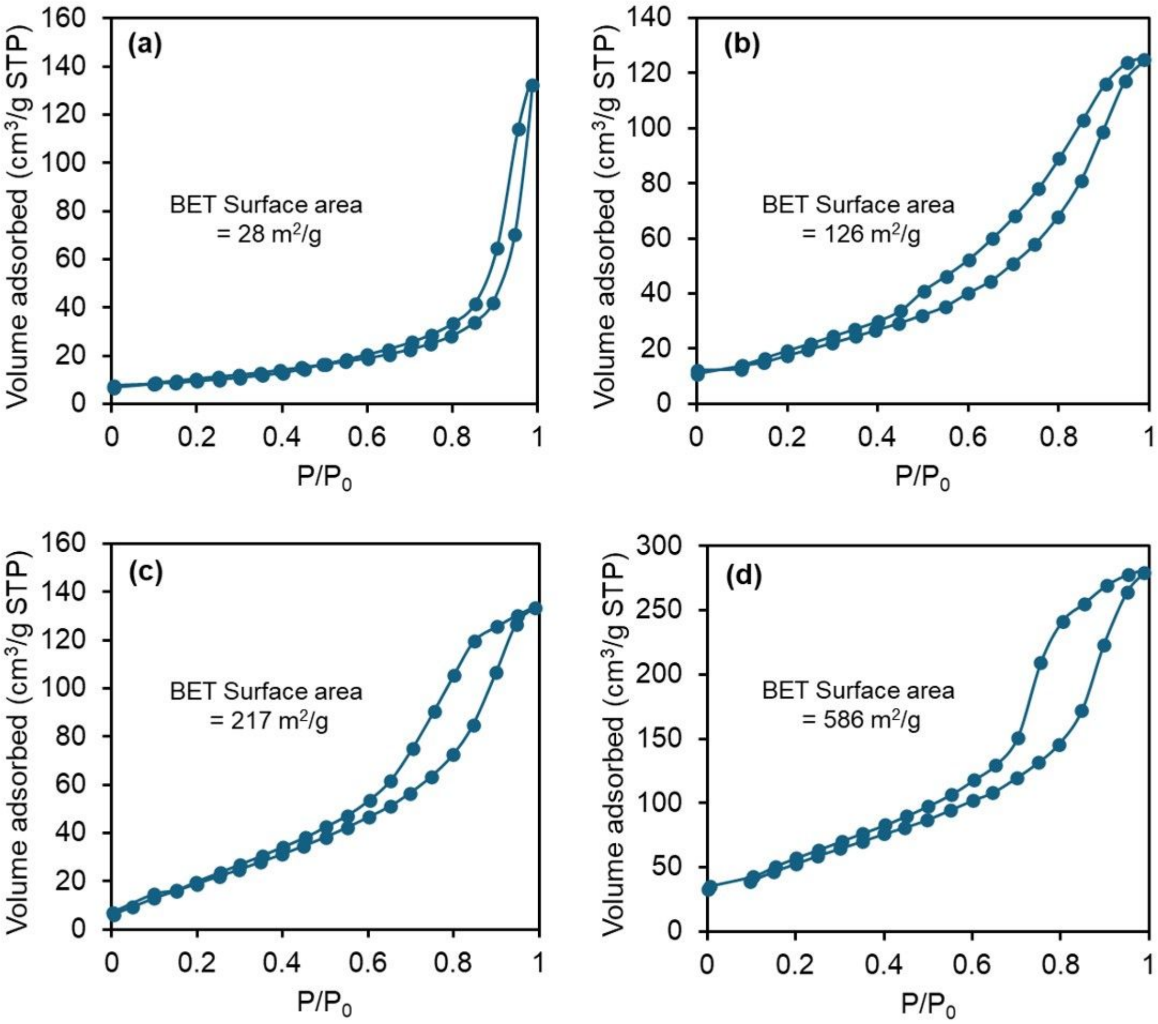

Figure S3. The BET surface area measurement of the as-intercalated graphite papers (a) without modification, (b) 1 min, (c) 3 min, and (d) 5 min after electrochemical intercalation.

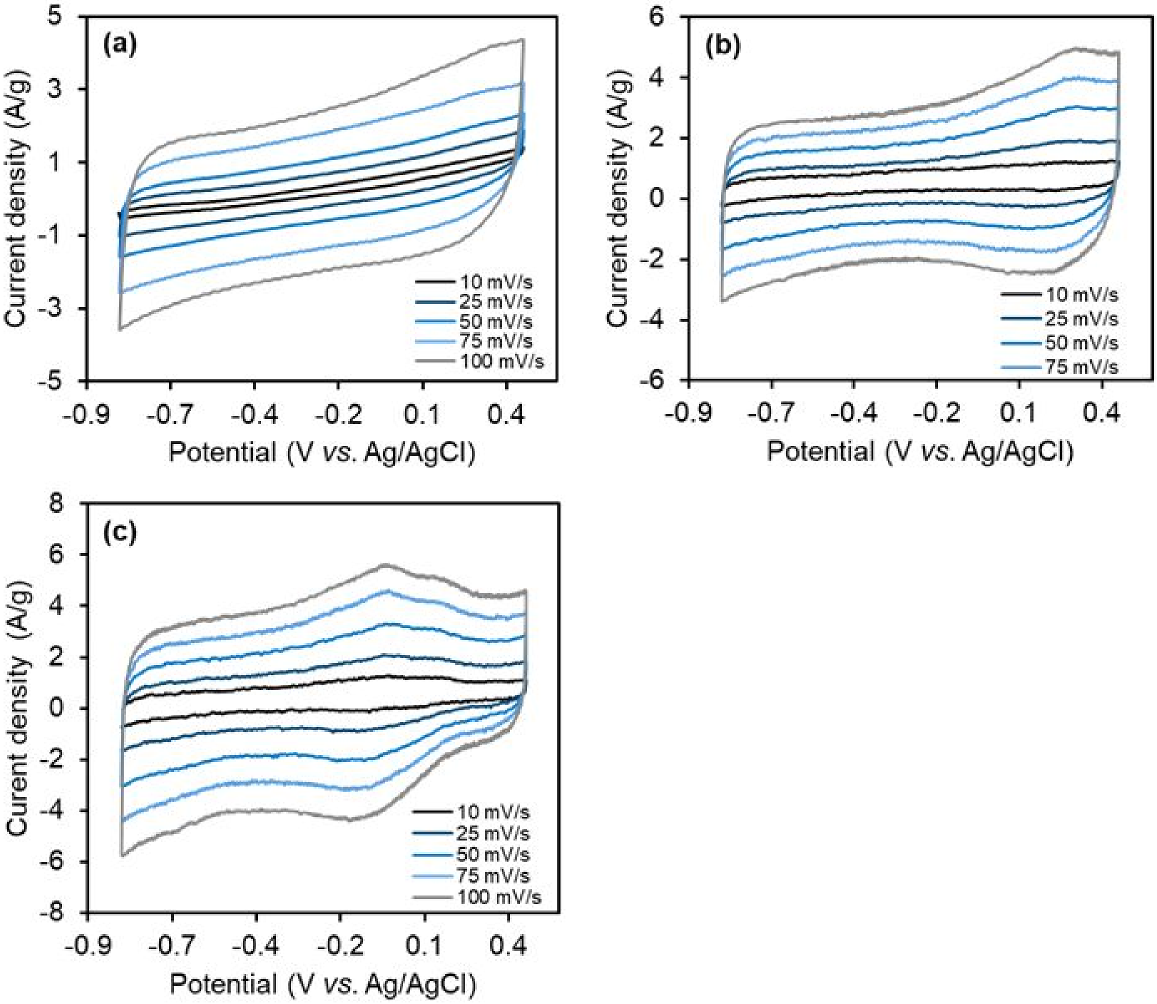

Figure S4. The CVs of the as-intercalated graphite papers at different scan rates. (a) 1 min, (b) 3 min, and (c) 5 min after electrochemical intercalation.

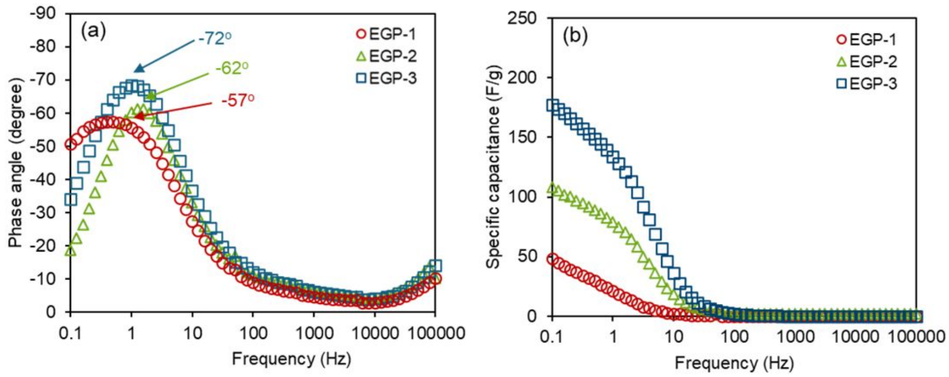

Figure S5. Capacitive analysis from EIS measurement of the treated graphite papers: (a) The Bode phase plot. (b) Specific capacitance as a function of frequency.

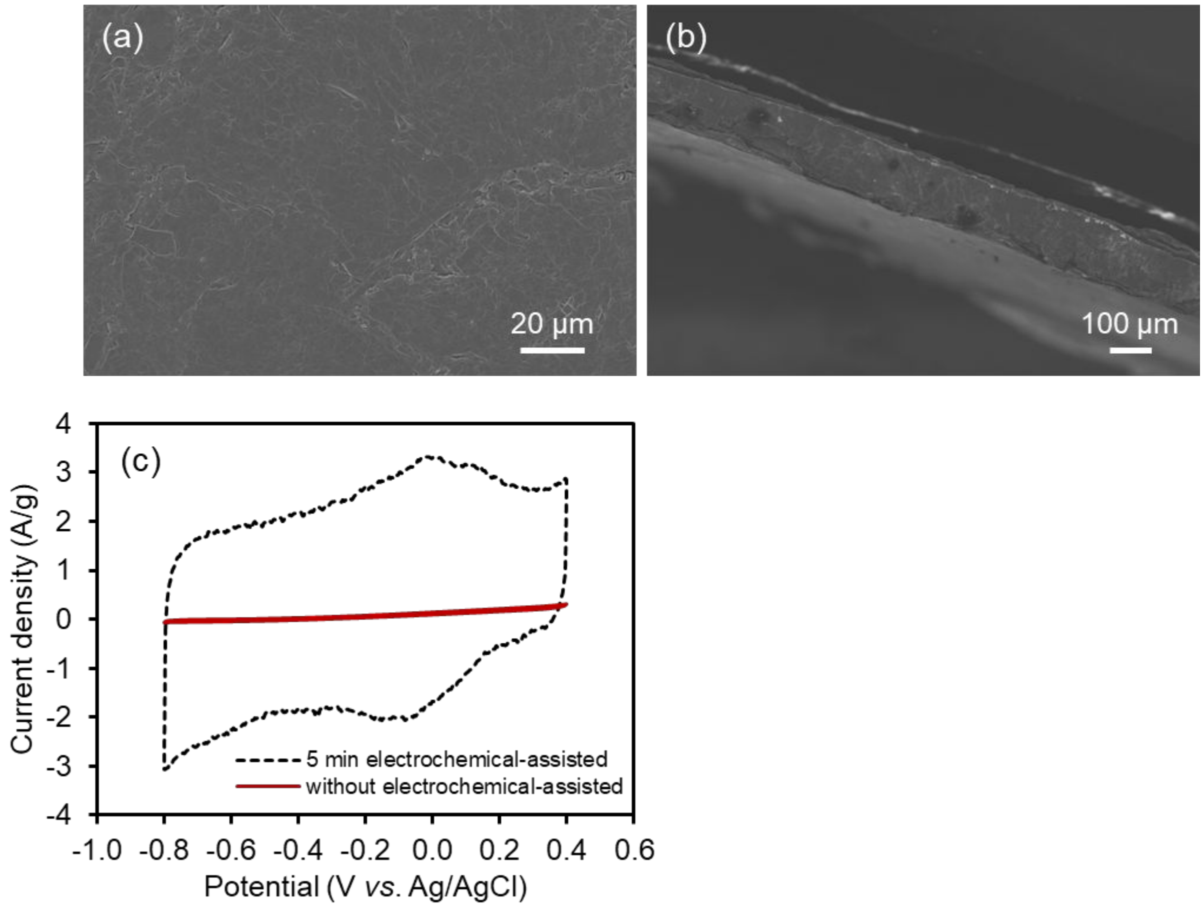

Figure S6. SEM images on graphite paper treated for 5 min without electrochemical assistance. (a) Top-view SEM image. (b) Cross-sectional SEM image. (c) Comparison of CV profiles at 50 mV s^{-1} for graphite treated for 5 min with and without electrochemical assistance.

Table S1. Comparison of various graphite exfoliation methods.

Material	Method	Treatment Solution	Potential (V) / Time	Capacitance (F/g)	Stability	Ref.
Highly ordered pyrolytic graphite	Electrochemical exfoliation	0.5 M H_2SO_4	+5 V for 600 s	N/A	N/A	[2]
Graphite plate	Electrochemical exfoliation	10 M H_2SO_4	+1 V for 10 min and ramped to +2 V for 20 min	113.2 F/g at 0.5 A/g	90% after 1000 cycles	[3]
Mechanically exfoliated graphite paper (MEGP)	Mechanical exfoliation, followed by sonication	DI water	Ultrasonication for 20 min	101.5 mF/cm^2 at 0.5 mA/cm^2 for MEGP/MWCNT@PPy	87.1% after 10000 cycles	[4]
Graphite rod (Partially exfoliated)	Electrochemical exfoliation	Mixed NaCl and DMSO aqueous solution	+ 5 V	N/A	N/A	[5]
Graphite rod to graphene	Electrochemical exfoliation	0.2 M $(NH_4)_2SO_4$	+ 10 V for 2 hrs	45 mF/cm^2 at mA/cm^2	92% after 5000 cycles	[6]
Partially exfoliated graphite paper	Hummer's method	Conc.H_2SO_4 with $NaNO_3$ and $KMnO_4$	-	0.923 F/cm^2 at 1 mA/cm^2	89% after 600 cycles	[7]
Graphite rod	Electrochemical exfoliation	0.1 M $(NH_4)_2SO_4$ in DI water with 1M H_2O_2 at 95 °C	+10 V for 2 hrs	N/A	N/A	[8]
Graphite flake	Microwave radiation	Graphite: $HClO_4$ 60%: Cu $(NO_3)_2$ and $KMnO_4$ (0.5:2:2:4 weight ratio)	Microwave heated at 800 W for 40 s	335.9 F/g at 0.5 A/g	94.4% after 3000 cycles	[9]
Graphite paper	Electrochemical exfoliation	0.1 M H_2SO_4	+10 V for 3 min	113 F/g at 10 mV/s	~100% after 500 cycles	This work

References

1. Good, R.J., *A Thermodynamic Derivation of Wenzel's Modification of Young's Equation for Contact Angles; Together with a Theory of Hysteresis1.* J. Am. Chem. Soc., 1952. **74**(20): p. 5041-5042.
2. Xia, Z., et al., *Electrochemical exfoliation of graphite in H_2SO_4, Li_2SO_4 and $NaClO_4$ solutions monitored in situ by Raman microscopy and spectroscopy.* Faraday Discuss., 2021. **227**(0): p. 291-305.
3. Wu, L., et al., *Powder, Paper and Foam of Few-Layer Graphene Prepared in High Yield by Electrochemical Intercalation Exfoliation of Expanded Graphite.* Small, 2014. **10**(7): p. 1421-1429.
4. Zhou, H., et al., *Mechanically exfoliated graphite paper with layered microstructures for enhancing flexible electrochemical energy storage.* Inorg. Chem. Front., 2022. **9**(9): p. 1920-1930.
5. Zhou, M., et al., *Few-layer graphene obtained by electrochemical exfoliation of graphite cathode.* Chem. Phys. Lett., 2013. **572**: p. 61-65.
6. Sevilla, M., G.A. Ferrero, and A.B. Fuertes, *Aqueous Dispersions of Graphene from Electrochemically Exfoliated Graphite.* Chem. Eur. J., 2016. **22**(48): p. 17351-17358.
7. Raghunandanan, A., et al., *Partially Exfoliated Graphite Paper as Free-Standing Electrode for Supercapacitors.* ChemistrySelect, 2018. **3**(18): p. 5032-5039.
8. Hossain, S.T. and R. Wang, *Electrochemical Exfoliation of Graphite: Effect of Temperature and Hydrogen Peroxide Addition.* Electrochim. Acta, 2016. **216**: p. 253-260.
9. Sykam, N., M. Ghosh, and G. Mohan Rao, *Exfoliated graphite containing metal oxides for high-performance pseudocapacitor applications.* J. Alloys Compd., 2018. **769**: p. 274-281.
